# Supporting Care Home Staff with Dementia and Behaviour Management Strategies

**DOI:** 10.1192/bjo.2026.11290

**Published:** 2026-06-30

**Authors:** Emily Pettifor, Caroline Watson, Nick Francis, Sarah Holmes

**Affiliations:** Kent and Medway Mental Health NHS Trust, Dartford, United Kingdom

## Abstract

**Aims:**

Care homes were noted to refer residents with Behavioural and PsychologicalSymptoms of Dementia (BPSD) without behavioural charts, which limited the development of appropriate care plans. Some referrals were also made to secondary mental health services before trial of local behavioural strategies. This project aimed for 80% of referrals to Kent and Medway Mental Health Trust for BPSD to include a medication chart and completed behavioural chart prior to assessment. A further aim was to design and deliver training on Dementia, BPSD and effective behavioural chart completion within two pilot care homes, with the longer-term intention of reducing referrals.

**Methods:**

A multidisciplinary team (MDT) mapped the referral pathway and collected baseline patient data, including whether behavioural charts were provided on referral, and chart quality from psychology and medical perspectives. A 3-hour MDT training package was developed, combining presentation, written materials and interactive case discussion. Topics included understanding Dementia, recognising behaviours experienced as challenging, communication strategies, use of behavioural charts with example walkthroughs, and approaches to supporting wellbeing and quality of life. Training was delivered on-site in two pilot homes, with staff encouraged to cascade learning. Trust referral screeners were also asked to request missing behavioural charts. Equivalent referral data was collected post-training.

**Results:**

Thirteen attendees provided feedback. All rated the training 10/10 for usefulness. Self-rated understanding of Dementia improved from 4.2/5 to 5/5, and confidence with completing behavioural charts improved from 6.8/10 to 9.8/10. Attendees included HCAs, Team Leaders and Managers. Qualitative comments highlighted learning around triggers for behaviours that challenge, completion of behavioural charts and behavioural strategies to potentially use for their residents with Dementia.

At baseline, 16 referrals for BPSD were accepted over four weeks in March, with two including behavioural charts. Requested charts from the two pilot homes scored low for structure (1.6/5) and clinical usefulness (1.4/5). After the training, five referrals were accepted over four weeks in June, none from the pilot homes. In the following two months, no new patients were taken on from one pilot home and two from the other, one accompanied by a completed behavioural chart. Although new patient numbers decreased, too few charts were therefore submitted to evaluate quality improvement.

**Conclusion:**

The training was well received and increased staff confidence. Wider rollout is recommended, though individual home-based delivery was resource-intensive. An annual centralised session may be more efficient.

